# Göran Tomson: scientists can help achieve sustainable development

**DOI:** 10.2471/BLT.18.030918

**Published:** 2018-09-01

## Abstract

Countries signed up to the 2030 Agenda for Sustainable Development face the challenge of achieving economic progress without damaging the environment or depleting natural resources. Göran Tomson tells Fiona Fleck how scientists can help.

*Q: Sustainable development was defined as “development that meets the needs of the present without compromising the ability of future generations to meet their own needs” in the 1987 report *Our common future*. What drew you to public health and sustainable development?*

A: When I started working as a paediatrician at a university hospital I was unaware of the importance of socioeconomic factors for children’s health and well-being. This was a gradual awakening for me. I was also influenced by my mother’s solidarity with those less fortunate and I was becoming increasingly aware of the appalling inequities globally. When I switched from clinical work to public health, my mentor Göran Sterky inspired me. My interest in sustainability is related to all of these issues and to the future of my grandchildren.

Q: The Sustainable Development agenda assumes that current efforts to achieve economic development are not sustainable. What forms of development are not sustainable and need to change?

A: We live in an age in which humans dominate the planet in an unprecedented way. The last few decades have seen major progress in development, with increased life expectancy and child mortality halved. However, we also see increasing inequities in access to health care and in health outcomes. Health is to a large extent determined by social factors. Moreover, global and national governance for health is weak. These problems call for transformative action. The fact that 193 governments committed themselves to the sustainable development agenda is an important recognition of these challenges, the complexities of addressing them and the fact that, unless we find a better balance between human needs and behaviour, the sustainability of our planet is threatened. Health and sustainable development should go hand in hand.

Q: How can we make this happen?

A: Take some of the sustainable development goals (SDG): SDG 5 calls on us to strive for gender equity, SDG 4 for better education, SDG 16 for more peaceful and inclusive societies with accountable institutions and SDG 3 for improved health and well-being for all. These goals are also interlinked. For example, we need to produce and distribute food with the smallest environmental footprint possible because our health depends on unpolluted environments, as well as good nutrition. This should not be difficult: the production of healthy food (especially unprocessed food items) often has a lower environmental impact than systems producing unhealthy food (ultra-processed items). The environmental approach is vital for human health, because our health depends on unpolluted environments and well functioning economic systems to provide safe drinking water, healthy and nutritious food, and recreational spaces for cognitive development and psychological benefits. Humans are affected by almost every aspect of the biosphere. Neglecting this fact will have grave consequences for our health.

“The environmental approach is vital for human health because our health depends on unpolluted environments.”

Q: What does a sustainable food system look like?

A: Sustainable diets are nutritionally beneficial, but also consider broader aspects of global sustainability. Switching to healthy plant-based diets with lower animal-source food content could reduce greenhouse gas emissions, thus reducing global warming, but also free land and provide more spaces for active transport (foot/cycle paths) and physical exercise. So freeing land has co-benefits for health and in particular for reducing noncommunicable diseases. With urbanization in many low- and middle-income countries, dietary patterns are rapidly changing with an increasing intake of processed food rich in fat and sugar. This is creating growing problems with diabetes, hypertension and obesity, resulting in an NCDs epidemic. We need to wind back this rapid change.

Q: Are there other examples of the lack of sustainability in our approach to health?

A: Antimicrobial resistance shows how antibiotics, a global good that should benefit everyone, are becoming ineffective. This is known as the tragedy of the commons. To avert this disaster, all sectors need to recognize what they can do to preserve the effectiveness of antimicrobials for the common good. High intensity farming, for example, is closely related to antimicrobial resistance. By 2030, the demand for meat, particularly in emerging economies such as China and India, is expected to increase antimicrobial use in animal husbandry by two-thirds. But while the routine antibiotics use in livestock production is an important factor in the emergence and spread of resistance, the major driver is overuse in the human health sector.

Q: Can you tell us about your work on antimicrobial resistance in Sweden?

A: I facilitated the development of the multidisciplinary course in 1987 entitled Medicines in Society with eight Asian teams held at the Karolinska Institutet. This caught the attention of a colleague from Management Sciences for Health in the United States of America and together with WHO we founded the International Network for Rational Use of Drugs that worked with teams from low and middle-income countries. In 2005, the Dag Hammarskjöld Foundation asked me to meet Otto Cars, who has championed efforts to contain antibiotic resistance in Sweden with the national programme, Strama. Cars understood that working within national borders was not sufficient, and that international work was also needed to protect Sweden. Back then, antibiotic resistance was not yet considered a global threat to modern medicine that hits resource-poor settings most. Now it is on many people’s agendas. That’s why we set up ReAct, a global antibiotic resistance advocacy network.

Q: What is the sustainable solution to the problem of antimicrobial resistance?

A: Countries need to develop animal husbandry practices that achieve high productivity without using antimicrobials for growth promotion or mass medication, while promoting rational consumption and distribution of these medicines for human health to preserve their effectiveness. Some countries have done this successfully. For example, Sweden’s national programme Strama does this by monitoring antibiotic use, setting prescription targets, surveillance of resistance trends, infection control and communication. Strama has achieved a sustained reduction of antibiotic use and lower levels of resistance for most bacterial species. Unless we take a One Health approach that addresses the animal, environmental and human spheres, we are doomed to failure. Another driver of antimicrobial resistance is the lack of access to effective antibiotics in many low- and middle-income countries. This also needs to be addressed.

Q: How helpful is it for countries to address the links between the SDGs?

A: It is essential. The potential synergies and trade–offs between the goals make interactions between the SDGs complex, but inevitable. Synergies are where efforts to achieve two or more goals support each other mutually, whereas a trade-off is when progress towards achieving one goal hinders progress in another. Some studies, including one on child health that SIGHT published with global health colleagues this year, anticipate more synergies than trade-offs for improving health overall. The paper suggests that multisectoral collaboration with the people who are implementing programmes is essential for further progress on child health. So by addressing [the links between] SDG1 on eradicating extreme poverty, SDG2 on ending hunger and malnutrition, SDG4 on free equitable education, SDG6 on water and sanitation, sustainable progress can be made on child health, covered by SDG3. We scientists need to communicate such SDG links so that they are better understood.

“We scientists need to communicate such SDG links so that they are better understood.”

Q: Can you give an example of goals that conflict?

A: Countries are using antibiotics to produce more meat, which supports SDG 2 on ending hunger, and there is widespread use of antibiotics in humans to cure diseases, supporting SDG3 on health. While these efforts are seen as promoting development, they lead to more antimicrobial resistance which undermines SDG3 on health. SDG12, on responsible consumption and production, is associated with several of these problematic trade-offs with other goals. According to a study by Pradhan et al. published in *Earth’s future* last year, SDG12 has negative correlations with 10 of the 17 goals (SDGs 1–7, 9, 10, 17).

Q: The lack of data was a barrier for tracking countries’ progress towards the Millennium Development Goals. How will progress be measured towards the SDGs?

A: National policymakers face the challenge of implementing the 2030 Agenda to achieve progress across economic, social and environmental dimensions of sustainable development. This requires a scientific analysis of the links between the SDGs, using a range of tools. In the child health study I mentioned, we used the SDG interactions framework scoring method, developed by colleagues from the Stockholm Environment Institute. Countries need to be able to monitor the core targets set for each goal, but also the so-called extended targets that are linked with the area in question, yet covered by other goals. Integrated policymaking is needed to take account of synergies and spillovers between all sectors. Integrated policy-making is when policymakers go beyond individual sectors, such as with SDG 3 on health, including links with SDG1 on poverty, SDG4 on education, SDG5 on gender and SDG16 on peaceful and inclusive societies, thus working across many sectors. The idea would be to leverage positive links, while reducing the negative effects of others.

Q: What can you and other scientists working on global health in Sweden do to support country efforts to achieve the goals?

A: Academic institutions in Sweden and elsewhere need to partner with countries and local research institutions to provide knowledge and evidence for governments and their implementing agencies. Providing scientific evidence alone is not enough for countries to achieve a transformation. That is why at SIGHT, for example, we would like our network to contribute by bringing scientists and other stakeholders including policymakers together so that they can better understand each other and collaborate for the global good.

